# Supplementary material for: An Analytic Orthotropic Heat Conduction Model for the Stretchable Network Heaters
Source: Micromachines (Basel). 2022 Jul 18;13(7):1133. doi: 10.3390/mi13071133 (PMC9318218; doi:10.3390/mi13071133)
Supplement: Supplementary file 1 [file micromachines-13-01133-s001.zip › micromachines-1791227-supplementary.pdf]

Supplementary Information

An Analytic Orthotropic Heat Conduction Model for the Stretchable Network Heaters

Zeqing He [1], Yingli Shi [2,*], Jin Nan [3], Zhigang Shen [4], Taihua Zhang [1] and Zhao Zhao [3,5,*]

[1] Aerospace Information Research Institute, Chinese Academy of Sciences, Beijing 100094, China; zqhe@aoe.ac.cn (Z.H.); zth@aoe.ac.cn (T.Z.)
[2] School of Materials and Energy, University of Electronic Science and Technology of China (USETC), Chengdu 610054, China
[3] Institute of Solid Mechanics, Beihang University (BUAA), Beijing 100191, China; nanjincn@163.com
[4] Beijing Key Laboratory for Powder Technology Research and Development, Beihang University (BUAA), Beijing 100191, China; shenzhg@buaa.edu.cn
[5] China Special Equipment Inspection and Research Institute, Beijing 100029, China
* Correspondence: yingli.shi@outlook.com (Y.S.); ouczhaozhao@163.com (Z.Z.)

Supplementary S1: Calculation of current density ratio in different paths of triangular and honeycomb heat source

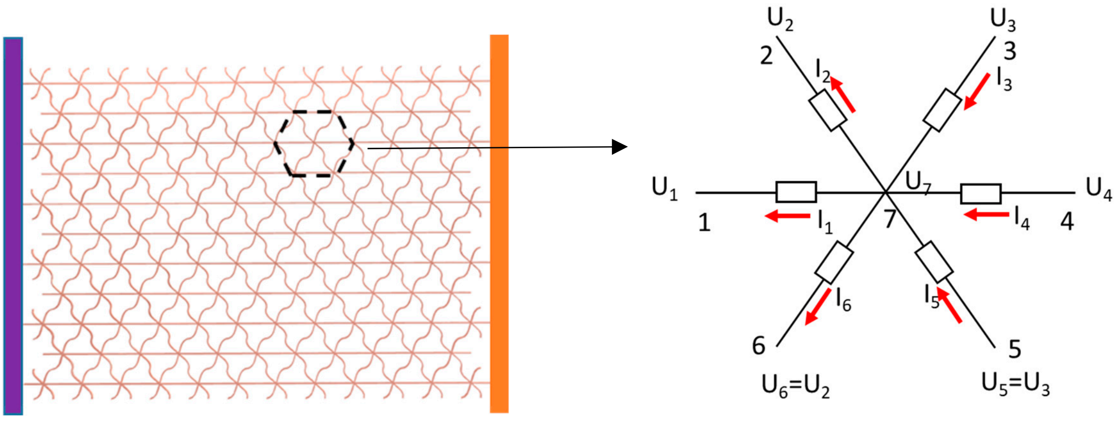

Figure S1. Configurations of the stretchable triangular heat source and the equivalent circuit.

The unit cell of the triangular network heater can be equivalent to the following circuit form. Based on the periodicity, I_3 and I_6 are equivalent ($I_3 = I_6$). Therefore, $U_3 - U_7 = U_7 - U_6$ can be obtained. Combined with $U_3 - U_7 = U_2 - U_1$, we can get $U_7 - U_1 = 2 (U_3 - U_7)$, which means $I_1 = 2 I_3 (I_2)$. Thus, the ratio of current density in the horizontal and oblique path is 2:1 from the equivalent circuit approach combined with the periodic conditions. Based on the Joule's law, the heat flux density ratio in the horizontal and oblique path should be 4:1.

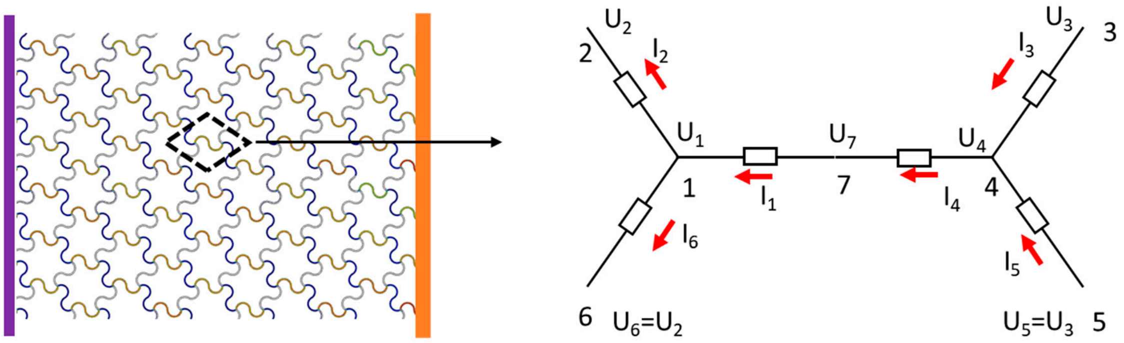

Figure S2. Configurations of the stretchable honeycomb heat source and the equivalent circuit.

The unit cell of the honeycomb network heater can be equivalent to the following circuit form. From the equivalent circuit, the currents satisfy the following relation: $I_1 = I_4 = I_3 + I_5 = I_2 + I_6$. Based on periodic geometry, the following relation holds: $U_2 = U_6$ and $U_3 = U_5$. Therefore, $I_2 = I_6$ and $I_3 = I_5$ can be obtained. To sum up, we can get the following relationship: $I_1 = I_4 = 2\ I_3 = 2\ I_5 = 2\ I_2 = 2\ I_6$. Thus, the ratio of current density in the yellow, blue and gray path is 2: 1: 1 from the equivalent circuit approach combined with the periodic conditions. Based on the Joule's law, the heat flux density ratio in the yellow, blue and gray path should be 4: 1: 1.

Supplementary S2: The meshing of 3D models and 3D simulation results for different type of heaters

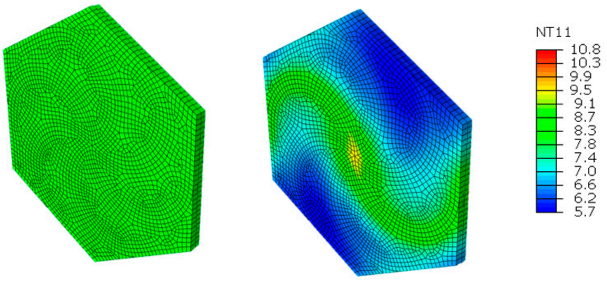

Figure S3. Meshing of 3D models and 3D simulation results for triangular heat source.

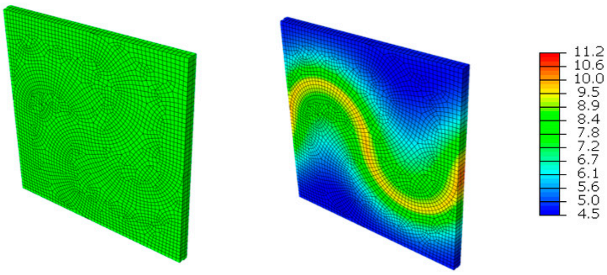

Figure S4. Meshing of 3D models and 3D simulation results for square heat source.

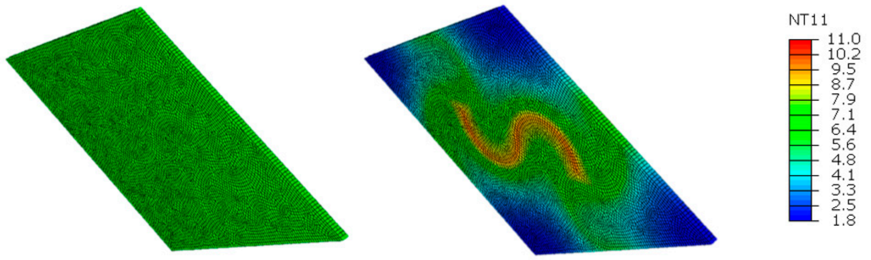

Figure S5. Meshing of 3D models and 3D simulation results for honeycomb heat source.
